# Inotropic agents use in patients hospitalized with acute decompensated heart failure: a retrospective analysis from a 22-year registry in a Middle-Eastern Country (1991–2013)

**DOI:** 10.1186/s12872-016-0223-5

**Published:** 2016-02-19

**Authors:** Amer H. S. Aljundi, Shaban F. K. Mohammed, Ashfaq Patel, Rajvir Singh, Abdulrahman Arabi, Hajar A. AlBinali, Jassim Al Suwaidi

**Affiliations:** Department of Clinical Pharmacology, Heart Hospital, Hamad Medical Corporation, P.O Box 3050, Doha, Qatar; Cardiovascular Research, Heart Hospital, Hamad Medical Corporation, Doha, Qatar; Cardiology and Cardiovascular Surgery, Heart Hospital, Hamad Medical Corporation, Doha, Qatar

**Keywords:** Inotropes, Acute decompensated heart failure, Predictors, Length of stay, Mortality

## Abstract

**Background:**

Data about the use of positive inotropic agents in patients hospitalized with acute decompensated heart failure (ADHF) is limited.

**Methods:**

The records of 8066 patients with ADHF who were hospitalized at Hamad Medical Corporation, Qatar from 1991 to 2013 were analyzed to explore demographics and clinical characteristics of the patients according to inotropic agents use.

**Results:**

Eight hundred fifty eight patients [10.6 %, 95 % CI (10 to 11.3 %)] received intravenous inotropic support. Patients receiving inotropes were more likely to be female and have preserved ejection fraction when compared to those not receiving inotropic agents. Comorbidities associated with higher likelihood of receiving inotropic treatment included acute myocardial infarction, chronic renal impairment, dyslipidemia, hypertension, obesity and hyperglycemia. Patient on inotropes were more likely to undergone percutaneous coronary intervention (PCI), intra-aortic balloon pump support and intubation. There were no differences in the mean plasma BNP and CK-MB levels between the 2 groups. Heart failure patients receiving inotropes also were more likely to have complications including ventricular tachycardia (2.0 % vs. 0.9 %, *p* = 0.003), prolonged hospital stay (8.0 vs. 5.0 days, *p* = 0.001), cardiac arrest (14.6 % vs. 3.2 %, *p* = 0.001) and in-hospital mortality (30.8 % vs. 9.1 %, *p* = 0.001). Over the study period there was an increase use of inotropic agents and decreased mortality rates.

**Conclusion:**

Inotropic use increased over the period whereas; female gender and conventional cardiac risk factors were predictors of inotropic agents use in the study.

## Background

Acute heart failure refers to a group of clinical syndromes due to either new-onset heart failure (HF) or decompensation of chronic HF with symptoms necessitating hospitalization, emergency department visits, or unscheduled medical attention [1]. Heart failure is a growing problem resulting in significant morbidity and mortality and is the most common cause of hospital admissions in the United States resulting in approximately 1.1 million hospitalizations annually [2–4].

Common causes of ADHF are coronary artery disease (CAD) or valvular abnormalities. However, most patients hospitalized with ADHF have a worsening of preexisting HF, while up to 20 % of patients have no prior diagnosis of HF [5].

Patients with ADHF typically present with symptoms that range in severity from mild volume overload to life-threatening cardiogenic shock and multi-organ failure [6]. A general goal in the management includes improved symptoms with hemodynamic stabilization and increased use of evidence-based therapies to reduce recurrent hospitalization and mortality.

Based on the recent American College of Cardiology/American Heart Association guidelines for the diagnosis and management of heart failure, patients who are refractory to diuretics should be managed with vasodilators as first-line therapy in the absence of symptomatic hypotension or systolic blood pressure of less than 90 mmHg. These guidelines also state that the use of intravenous positive inotropic agents is a valid option to improve contractility for hospitalized patients with heart failure who have evidence of low ejection fraction, hemodynamic compromise, cardiogenic shock, or decreased organ perfusion [7]. Of note, the clinical practice guidelines of the Heart Failure Society of America [8] and the European Society of Cardiology [9] have similar recommendations to that of the American College Cardiology/American Heart Association. Positive inotropic agents, on the other hand, have not been shown to reduce length of hospital stay or mortality when added to standard care [10]. Additionally, milrinone and dobutamine have also been shown to be proarrhythmic and cause more symptomatic hypotension in randomized controlled trials compared to standard care [11]. Several observational studies including that of the Acute Decompensated Heart Failure National Registry (ADHERE) have also shown increases in mortality with inotropes when compared with vasodilator therapy [12]. However, data about trends of inotropic agents use in ADHF patients over a period of time is lacking.

We analyzed predictors of overall use of positive inotropic agents and associated outcomes in patients hospitalized with acute decompensated heart failure over 22-years period.

## Methods

- Study setting
  This study was conducted in Heart hospital, Doha, Qatar. Heart Hospital is committed to deliver safe, high quality care in cardiology and cardiothoracic surgery for the residents of Qatar, nationals and expatriates where more than 95 % of cardiac patients are being treated in the Hospital, making it an ideal center for observational-based studies. The state of Qatar is located in the Arabian Peninsula having approximately 1.7 million population in 2010 most of them being Arab (40 %) followed by Indians (18 %), Pakistani (18 %), Iranian (10 %) and others (14 %). In the past decade, cardiovascular diseases were the leading cause of morbidity and mortality in the country. Database for patients admitted to the hospital are maintained electronically and registered at the cardiology department since January 1991. Based on these records, patients hospitalized due to ADHF between 1991 and 2013 were retrospectively evaluated. Study (#11355) was exempted for review from Institutional Review Board (IRB), MRC, Hamad Medical Corporation (HMC) due to its retrospective nature and study did not contain identifiable private information and were not collected by investigators through intervention or interaction with individuals.
  The Framingham criteria were used for the diagnosis of heart failure. The concurrent presence of either 2 major criteria or 1 major and 2 minor criterion was required to establish a diagnosis of HF [13]. Major criteria included paroxysmal nocturnal dyspnea, neck vein distention, weight loss of 4.5 kg in 5 days in response to treatment, acute pulmonary edema, rales, hepatojugular reflux, S3 gallop, central venous pressure greater than 16 cm water, circulation time of 25 s, radiographic cardiomegaly, pulmonary edema, visceral congestion, or cardiomegaly at autopsy. Minor criteria on the other hand included nocturnal cough, dyspnea on ordinary exertion, a decrease in vital capacity by one third the maximal value recorded, pleural effusion, tachycardia (heart rate of at least 120 beats per minute), or bilateral ankle edema that are not attributed to any other medical condition (e.g., cirrhosis, ascites, nephrotic syndrome) [14–17]. Inotropic support is defined as the use of adrenergic agonists and phosphodiesterase III (PDE) inhibitors. Doses of inotropic support were defined as the use of dopamine ≥ 5 μg/kg/min; any dose of epinephrine, norepinephrine, dobutamine, or milrinone. On the other hand, definition of acute myocardial infarction (MI) in this study was consistent to the World Heart Organization criteria. Bedside transthoracic echocardiography (TTE) was used to measure EF at admission to coronary intensive care unit. The presence of diabetes mellitus was based on documentation in the patient’s previous or current medical record. The diagnosis of hyperlipidemia was made by a fasting cholesterol >5.2 mmol⁄L in the patient’s medical record or any history of treatment of hyperlipidemia by the patient’s physician. Chronic renal impairment was defined as creatinine >1.5 upper normal range. Presence of hypertension was determined by any documentation in the medical record or treatment by the patient’s physician.
- Statistical analysis
  Descriptive statistics were calculated for all variables of interest. Patient characteristics were in the form of mean ± standard deviation (SD) and frequency with percentages for interval and categorical variables respectively. The frequencies of categorical variables were compared using chi-square test while comparisons were based on independent student *t*-test for interval data. To determine the patient characteristics that were associated with the use of inotropic agents, variables including age, sex, diabetes mellitus, hypertension, obesity, dyslipidemia, chronic renal impairment, ejection fraction, and prior MI were considered for multivariate logistic regression analysis using enter method. Adjusted odds ratios (ORs) and corresponding 95 % confidence intervals (CIs) for each factor were reported. All tests were 2-tailed and statistical difference was defined as a *p* value < =0.05. All data analyses were carried out using the Statistical Package for Social Sciences version 19.0 (SPSS, IBM, Armonk, NY).

## Results

The study included 8066 patients, of whom 858 patients received inotropic agents. Baseline characteristics of the study population hospitalized with ADHF are displayed in Tables 1 and 2. The study patients were racially diverse; fewer patients in the inotropic group were Asians but more Middle-Eastern Arabs. Patients receiving inotropes were more likely to be female (38.9 % vs. 33.1 %, *p* = 0.001), older age (63 vs. 62 years, *p* = 0.004), with chronic renal impairment (18.4 % vs. 8.5 %, *p* = 0.001), on dialysis (1.9 % vs. 0.3 %, *p* = 0.001), dyslipidemic (11.2 % vs. 5.5 %, *p* = 0.001), hypertensive (69.3 % vs. 56.9 %, *p* = 0.001), obese (8.7 % vs. 6.1 %, *p* = 0.003) and have diabetes mellitus (66.3 % vs. 57.1 %, *p* = 0.001) at the time of admission. The inotropes group was also more likely to have elevated troponin levels (40.8 % vs. 25.9 %, *p* = 0.001) and to present with both STsegment elevation (8.6 % vs. 4.3 %, *p* = 0.001) and non-ST-segment elevation (6.2 % VS. 3.6 %, *P* = 0.001) myocardial infarction when compared to the non-intropes group. The inotrope group was also more likely to undergo percutaneous coronary revascularization (2.0 % vs. 0.7 %, *p* = 0.001), and have intra-aortic balloon pump support (2.6 % vs. 0.4 %, *p* = 0.001). Interestingly, patients in the no inotropic support group were more likely to have lower LV ejection fraction (LVEF ≤ 40 %; 69.6 % vs. 59.2 %, *p* = 0.001) and more likely to develop atrial fibrillation (10.1 % vs. 6.2 %, *p* = 0.001) when compared to those who received inotropic agents. There were no significant differences between the 2 groups in regards to the mean plasma BNP levels (4195 ± 7450 vs. 3090 ± 7650) and CKMB levels (61 ± 192 vs. 74 ± 559) (*p* = 0.22 and 0.60 respectively).

**Table 1:** Heart Failure Patient Characteristics and Comorbidities

Variable	Inotropes	No Inotropes	*p* Value
Demographics			
Total number, n (%)	858 (10.6)	7208 (89.4)	
Age, mean (SD)	63 ± 12	62 ± 12	0.04
Male, n (%)	419 (61.1)	4270 (66.9)	0.001
Middle-Eastern Arabs	674 (78.6)	5472 (73.7)	
South Asians	164 (19.1)	1562 (21.7)	
Others	20 (2.3)	174 (2.4)	0.001
Patient Characteristics, n (%)			
Smoker	141 (16.4)	1043 (14.5)	0.12
Chronic renal impairment	158 (18.4)	612 (8.5)	0.001
Dyslipidemia	96 (11.2)	394 (5.5)	0.001
Hypertension	595 (69.3)	4100 (56.9)	0.001
Diabetes Mellitus	569 (66.3)	4115 (57.1)	0.001
Obesity	75 (8.7)	443 (6.1)	0.003
Menopause	34 (4)	206 (2.9)	0.07
Labs, mean (SD)			
Hemoglobin (g/dl)	11.8 ± 5	12 ± 4.9	0.04
Fasting blood sugar, mmol/L	11 ± 16	12 ± 48	0.81
Serum creatinine, mmol/L	164 ± 127	131 ± 265	0.01
BNP, (pg/ml)	4195 ± 7450	3090 ± 7650	0.22
Cholesterol, mmol/L	4.3 ± 1.5	4.4 ± 6	0.57
Low density lipoprotein (LDL), mmol/L	2.46 ± 1.0	2.9 ± 9.8	0.42
High density lipoprotein (HDL), mmol/L	1.2 ± 1.6	1.6 ± 1.9	0.63
Triglycerides, mmol/L	1.6 ± 1.3	1.6 ± 7.8	0.98
Creatinine kinase -MB, μ/L	61 ± 192	74 ± 559	0.6
Labs, n (%)			
Troponin positive	350 (40.8)	1866 (25.9)	0.001
Cardiac History, n (%)			
Admission ejection fraction ≤ 40 %	200 (59.2)	1305 (69.6)	0.001
Rheumatic heart disease	3 (0.3)	43 (0.6)	0.36
ST-elevation myocardial infarction (STEMI)	74 (8.6)	309 (4.3)	0.001
Non-STEMI (NSTEMI)	53 (6.2)	256 (3.6)	0.001
Unstable angina	64 (7.5)	421 (5.8)	0.06
Cardiomyopathy	125 (14.6)	860 (11.9)	0.03
Pulmonary hypertension	12 (1.4)	80 (1.1)	0.45
Valvular Heart Disease, n (%)			
Mitral regurgitation	44 (5.1)	373 (5.2)	0.95
Aortic regurgitation	11 (1.3)	106 (1.5)	0.66

**Table 2:** In-hospital Procedures and Complications

Variable	Inotropes	No Inotropes	*p* Value
Procedures, n (%)			
Intraaortic balloon pump (IABP)	22 (2.6)	28 (0.4)	0.001
Swan-Ganz catheterization	47 (5.5)	82 (1.1)	0.001
Percutaneous coronary intervention (PCI)	17 (2)	54 (0.7)	0.001
Coronary artery bypass grafting (CABG)	61 (7.1)	652 (9)	0.06
Intubation	58 (6.8)	76 (1.1)	0.001
Dialysis	16 (1.9)	22 (0.3)	0.001
Complications, n (%)			
Cerebrovascular accident (CVA)	4 (0.5)	39 (0.5)	0.78
Left bundle branch block (LBBB)	29 (3.4)	223 (3.1)	0.65
Supraventricular tachycardia (SVT)	3 (0.3)	13 (0.2)	0.3
Ventricular tachycardia	17 (2)	65 (0.9)	0.003
Atrial fibrillation	53 (6.2)	726 (10.1)	0.001
Ventricular fibrillation	5 (0.6)	10 (0.1)	0.004
Cardiac arrest	125 (14.6)	232 (3.2)	0.001
In-hospital mortality	193 (30.8)	407 (9.1)	0.001
Complications, mean (SD)			
Length of CCU stay	5 ± 7	4 ± 5	0.02
Total days hospitalized	8 ± 34	5 ± 103	0.001

### Outcome

Patients on inotropes group had more cardiovascular complications including; ventricular tachycardia (2.0 % vs. 0.9 %, *p* = 0.003), prolonged hospital stay (8.0 vs. 5.0 days, *p* = 0.001), cardiac arrest (14.6 % vs. 3.2 %, *p* = 0.001) and in-hospital mortality (30.8 % vs. 9.1 %, *p* = 0.001) (Table 2).

### Trends

Inotropes use over the study period significantly increased; 4.6, 9.0, 9.9 14.2 and 17.6 % (*p* = 0.001) in 1991–95, 1996–2000, 2001–05, 2006–2010 and after 2010 years respectively (Table 3).

**Table 3:** Trend of inotropic support in Heart Failure patients

Trend in years	1991–95	1996–2000	2001–05	2006–10	>2010	*P* value
Inotropic	65(4.6 %)	159(9.0 %)	201(9.9 %)	277(14.2 %)	154(17.6 %)	0.001
Support						

The average mortality rates in patients with ADHF receiving inotropic agents over 22 years was variable but decreased recently; 17.7, 27.1, 29.2, 18.8, and 7.3 % (*p* = 0.001) in 1991–95, 1996–2000, 2001–05, 2006–2010 and after 2010 years respectively (Table 4).

**Table 4:** Trend of mortality in Heart Failure patients

Trend in years	1991–95	1996–2000	2001–05	2006–10	>2010	*P* value
Mortality	17.7 %	27.1 %	29.2 %	18.8 %	7.3 %	0.001

### Multivariate analysis

Table 5 shows multivariate logistic regression analysis; the adjusted odds of being treated with any positive inotropic agent were highest in patients who were a history of hypertension (OR: 1.66, 95 % CI: 1.10 to 2.5) or having chronic renal impairment (OR: 1.76, 95 % CI: 1.19 to 2.6) or preserved ejection fraction (OR: 1.92, 95 % CI: 1.27 to 2.9) after adjusting age, gender, hyperglycemia, obesity, ethnicity and old myocardial infraction.

**Table 5:** Multivariate predictors of Inotropes in Patients Hospitalized with ADHF

Variable	Adjusted OR	95 % CI	*p* value
Age	0.99	0.98–1.01	0.33
Male	0.90	0.64–1.27	0.55
Diabetes Mellitus	1.04	0.72–1.50	0.84
Hypertension	1.66	1.10–2.50	0.02
Obesity	1.06	0.66–1.70	0.81
Chronic renal failure	1.76	1.19–2.60	0.005
Ejection fraction >40	1.92	1.27–2.90	0.002
Middle-Eastern Arabs	1.10	0.76–1.58	0.62
Old myocardial infarction	1.19	0.82–1.71	0.36

In terms of evidence-based discharge medications, more patients in the inotropic support received beta-blockers at discharge and but ACE inhibitors when compared to patients who did not receive inotropes agents (Table 6).

**Table 6:** Medication Use at Discharge

Variable	Inotropes	No Inotropes	*P* Value
ß-blocker	275 (32.1)	1908 (26.5)	0.001
ACE/ARB^a^	321 (37.4)	4346 (60.3)	0.001
Calcium channel blockers	352 (41)	768 (10.7)	0.001
Digoxin	28 (3.3)	389 (5.4)	0.008
Amiodarone	53 (6.2)	394 (5.5)	0.39
Frusemide	545 (63.5)	6012 (83.4)	0.001
Spiranolactone	35 (4.1)	456 (6.3)	0.009

^a^ACE/ARB: Angiotensin converting enzyme inhibitors/Angiotensin receptor blockers

## Discussion

This 22-year observational study of patients hospitalized with HF demonstrates 10.6 % need to use inotropic agents. Patients who received inotropic agents were older, more likely to be female, local Arabs rather than Asians with worse cardiovascular risk profile. Inotropic agents group were more likely to present with acute coronary syndrome when compared to the non-inotropic agents group. Interestingly, low left ventricular ejection fraction and BNP were not predictors of its use.

The current observation of inotropic agents use is consistent with that reported from the ADHERE Registry, which reported ≈ 10 % use of inotropic agents in patients hospitalized for ADHF in the United States. It is also consistent with other reports in the literature, which varied from 3 to 12 % [4, 12, 18]. Interestingly we report significant increase of inotropic support over the 22 years period, which may suggest hospitalization of “sicker” patients, while patients with less severe heart failure are increasingly managed in the outpatient setting.

### Predictors of inotropic agents use

Our study found that the strongest predictors of inotrope use were hypertension and chronic renal impairment. Based on data from ADHERE Registry, about 9.6 % of patients hospitalized for ADHF in the United States received an intravenous inotrope (either milrinone or dobutamine). Such patients tended to have more severe disease, including lower blood pressure, lower ejection fraction, and higher blood urea nitrogen. The use of inotropes in the context of normal ejection fraction in our study is also striking because this signifies an overuse of inotropic medications, a fact that is consistently discouraged. Based on (ADHERE) Registry, out of more than 150,000 patients, fewer than 3 % had a systolic BP of <90 mmHg while approximately 50 % had a preserved systolic function. [19] However, about 14 % of the patients in ADHERE were treated with an intravenous inotropic agent indicating an overuse of such agents. Many studies have revealed that the use of inotropic agents may be associated with increased mortality, especially in patients with preserved left ventricular function. Therefore, it is recommended that positive inotropes only be used in patients who require inotropic support for low cardiac output.

However, better LVEF observed in inotropic group in our study may be explained by the susceptibility of these patients to a sudden drop in ejection fraction, even though small, compared to patients pre-conditioned to chronic low ejection fraction. Moreover, our study patients were not stratified based on other reversible conditions like sepsis or infection that can lead to an increase in demand and where the use of inotropes is assumed to improve outcomes.

One of the strongest predictors of inotrope use in our study was hypertension. Large multicenter registries revealed that about 70 % of patient hospitalized with ADHF have fluid overload, normal to elevated blood pressure with relatively preserved end-organ function, and no evidence of cardiogenic shock [4, 6, 20]. Therefore, the use of inotropes, with the main goal of improving end-organ perfusion, in such patients is not rational. Of note, for the majority of patients with ADHF with normal or elevated systemic blood pressure, there are no data to support a role for inotropic therapy. In this context, it is worth mentioning that 9 % of patients in the ADHERE Registry received a positive inotrope (dobutamine or milrinone), although only 2 % of patients were hypotensive. This means that more than 75 % of patients had no clinical indication for their use signifying an overuse of such medications.

Based on the European Society of Cardiology guidelines for the diagnosis and treatment of acute HF, patients with ADHF can be classified into one of six groups based on the clinical presentation [9]. The first 3 categories of patients comprise more than 90 % of ADHF presentations. These patients normally have relatively preserved left ventricular function with signs and symptoms of pulmonary edema and hypertension. Regardless, these findings emphasize the need for well-designed prospective trials to further establish the appropriate use of intravenous inotropic therapy in this population of patients.

The presence of renal dysfunction is relatively common in heart failure and can reduce the adequacy of diuresis. Renal dysfunction in our analysis was a strong predictor of inotropic use. This observation can be explained by the fact that when increases in intravenous boluses or infusions of loop diuretics and addition of metolazone does not lead to an adequate diuretic response, facilitation of diuresis may be enhanced by inotropic therapy by presumably improved renal blood flow through modest increases in cardiac output [21]. Moreover, at our institution, low-dose dopamine is used in combination with diuretic therapy, on the supposition that it can increase renal perfusion and subsequent diuresis. In general, patients with clinical evidence for end-organ hypoperfusion are mostly referred to as having a low output state. While hypotension is classically considered a powerful predictor of this hemodynamic profile, clinical exam findings may inaccurately identify such patients who may not even present with hypotension [22] Of note, patients with long standing heart failure, low output states can be associated with more subtle clinical findings, such as nausea, abdominal pain, slowed mentation, and fatigue [23]. An extremely important clinical finding indicating impaired end-organ perfusion is worsening renal function (so- called cardiorenal syndrome), a finding that usually occurs after initiating intravenous loop diuretics [24]. More recent data suggest that many patients with cardiorenal syndrome have a relatively preserved cardiac output and hypertension making them amenable to treatment with inotropes [25].

### Outcomes

Our analysis provides data that focus on outcomes related to the mortality of patients treated with inotropic medications. This consideration is important when comparing our results to other published studies evaluating mortality. Inotropic therapy in certain studies has been shown to have detrimental effects [5, 10, 26–36], although another meta-analysis was unable to confirm this view [37]. In ADHERE registry; more than 65,000 patients from 263 centers across the United States admitted with heart failure were retrospectively analyzed. Data were utilized to describe the management trends and outcomes of those patients. Based on the registry, use of short-term vasodilator therapy was associated with significantly lower in hospital mortality when compared to positive inotropic treatment. A propensity score matching was used to adjust for confounding factors when comparing patients in both arms. Similarly, the impact of intravenous inotropic therapy on mortality in patients hospitalized with ADHF has been validated in the Outcomes of a Prospective Trial of Intravenous Milrinone for Exacerbation of Chronic Heart Failure (OPTIMECHF) study. In this placebo-controlled, randomized, double-blind clinical trial, the use of intravenous milrinone when compared to placebo was associated with a higher rate of early treatment failure, sustained hypotension (10.7 % vs. 3.2 %, *p* < 0.001), and new onset atrial fibrillation or flutter (4.6 % vs. 1.5 %, *p*=0 .004) [10]. There were no improvements in total days hospitalized, symptomatic relief, or mortality with milrinone compared with placebo. Our analysis revealed that intravenous inotrope use was associated with a significant increase in the risk of mortality. This risk association could not be attributed to the use of inotropic medications. Therefore, controlling for other potential risk factors as possible using a propensity score–adjusted multivariable analysis would be better utilized to conclude a significant worse prognosis with the independent use of inotropes compared with the other group. Of note, in terms of predictors of in-hospital mortality, of the 39 variables examined in ADHERE, the strongest predictor was a blood urea nitrogen (BUN) of 43 mg/dL or higher. This was followed by a systolic blood pressure of less than 115 mmHg and a SCr of 2.75 mg/dL or greater.

In-hospital mortality in the inotrope-treated patients in our study was close to 31 %. In ADHERE, patients treated with Inotrops had a high mortality rate (19 %) than all other non-inodilator-treated patients (14 %) or patients with preserved ejection fraction not treated with inotropes (2 %) [12]. Proposed mechanisms of increased mortality with inotropes in our study include in-hospital events such as tachyarrhythmias or subclinical myocardial ischemia and apoptosis through increased sympathetic stimulation and intramyocardial calcium accumulation with the use of inotropes. Additionally, many studies have revealed that the use of inotropic agents may be associated with increased mortality, especially in patients with preserved left ventricular function, which is also consistent with our observations.

The use of intravenous inotropes increased over the last years despite the absence of a reasonable indication with a trend in increasing mortality.

In addition to evaluating the in-hospital mortality rate, our study also considered other relevant end points, such as complications of inotropic support, length of CCU stay, and length of hospital stay. As opposed to the results of (OPTIME-CHF), our analysis revealed lower incidence of atrial fibrillation in patients treated with inotropic support. This fact is interesting as patients in this group had more cardiovascular risk factors for developing atrial fibrillation. However, patients on inotropes had more episodes of ventricular tachycardia and cardiac arrest; associated rhythm was ventricular fibrillation. We infer that to the use of inotropic agents that are known to trigger ventricular tachyarrhythmia as such complications have been confirmed in randomized trials of inotropic agents versus placebo or vasodilator medications used for heart failure [38, 39]. Therefore, the use of such medications warrant close monitoring and correction of any cause that can contribute to development of arrhythmias.

Length of CCU stay and overall hospital stay was longer in patients treated with inotropic medications group. This result is consistent with other registries. For example, patients who received inotropic agents in ADHERE had more than double the length of stay (12.9 vs. 5.8 days; *P* < 0.0001) compared with those who were not treated with inotropes. Moreover, in a subgroup analysis of ADHERE, patients with preserved systolic function treated with inotropic agents had longer length of hospital stay (12.9 vs. 5.8 days; *P* < 0.0001) and more than nine-fold increase in mortality rate (19 vs. 2 %; *P* < 0.0001) compared with patients who did not receive inotropes [40].

One of the important goals in the management of patients with ADHF is to implement the use of evidence-based therapies to reduce recurrent hospitalization and mortality, which include angiotensin converting enzyme (ACE) inhibitors and beta-blockers [41]. According to our analysis, more patients in the inotropic support received beta-blockers but less ACE inhibitors compared to no inotropes group (Table 6). This can be explained by the fact that patients in the inotropic support had more tachy-arrhythmias that augmented the use of beta-blockers. . Of note, ACE inhibitors provide rapid improvement in hemodynamics and renal function that may facilitate the subsequent initiation of betablockers.

### Limitations of the study

Our study had several limitations. First, it is historical and observational in design with its inherent limitations including missing data or measurement errors. Additionally, patients were not randomly assigned to the treatment groups and, as a result, both groups differed in demographics and disease-associated factors related to the outcomes of interest. Because it is not possible to identify and adjust for all possible factors, the differences between patients in both group and the influences of other nonmeasured factors cannot be totally excluded. However, we controlled for between-group differences through multivariate analysis that took into consideration differences in baseline demographics and disease-associated factors related to the predictors of inotropes use. We also did not categorize patients based on the inotropic medication given. The study design cannot also overcome a bias from the inclusion of a select group of severe patients who may have been channeled to the inotropic support group with subsequent worse outcomes. Additionally, our database did not include clinical data of left ventricular ejection fraction and BNP throughout 22 years, which are important determinants of inotrope use and thus may contribute to improved risk adjustments.

## Conclusion

Conventional cardiac risk factors predict the use of inotropic support in heart failure patients. However, there was some overuse of inotropes in patients with hypertension and preserved ejection fraction. Inotopic agents use increased over the years.

## Abbreviations

ADHF: acute decompensated heart failure
BNP: brain natriuretic peptide
CK-MB: Creatinine kinase-MB fraction
PCI: Percutaneous coronary intervention
MI: myocardial infarction
ADHERE: Acute Decompensated Heart Failure National Registry

## References

[1] Felker GM, Adams KF, Konstam MA, O’Connor CM, Gheorghiade M (2003). The problem of decompensated heart failure: Nomenclature, classification, and risk stratification. Am Heart J.

[2] Najafi F, Jamrozik K, Dobson AJ (2009). Understanding the ‘epidemic of heart failure’: a systematic review of trends in determinants of heart failure. Eur J Heart Fail.

[3] Roger VL, Go AS, Lloyd-Jones DM, Adams RJ, Berry JD, Brown TM (2011). Heart disease and stroke statistics-2011 update: a report from the American Heart Association. Circulation.

[4] Adams KF, Fonarow GC, Emerman CL, LeJemtel TH, Costanzo MR, Abraham WT (2005). Characteristics and outcomes of patients hospitalized for heart failure in the United States: rationale, design, and preliminary observations from the first 100,000 cases in the Acute Decompensated Heart Failure National Registry (ADHERE). Am Heart J.

[5] Joseph SM, Cedars AM, Ewald GA (2009). Acute decompensated heart failure: contemporary medical management. Tex Heart Inst J.

[6] Gheorghiade M, Zannad F, Sopko G, Klein L, Piña IL, Konstam MA (2005). Acute heart failure syndromes: Current state and framework for future research. Circulation.

[7] Hunt SA, Abraham WT, Chin MH, Feldman AM, Francis GS, Ganiats TG (2009). 2009 focused update incorporated into the ACC/AHA 2005 guidelines for the diagnosis and management of heart failure in adults. A report of the American College of Cardiology Foundation/American Heart Association Task Force on Practice guidelines developed in collaboration with the International Society for Heart and Lung Transplantation. J Am Coll Cardiol.

[8] Lindenfeld J, Albert NM, Boehmer JP, Collins SP, Ezekowitz JA, Heart Failure Society of America (2010). HFSA 2010 comprehensive heart failure practice guideline. J Card Fail.

[9] Dickstein K, Cohen-Solal A, Filippatos G, McMurray JJ, Ponikowski P, PooleWilson PA (2008). ESC guidelines for the diagnosis and treatment of acute and chronic heart failure 2008: the Task Force for the Diagnosis and Treatment of Acute and Chronic Heart Failure 2008 of the European Society of Cardiology. Developed in collaboration with the Heart Failure Association of the ESC (HFA) and endorsed by the European Society of Intensive Care Medicine (ESICM). Eur J Heart Fail.

[10] Cuffe MS, Califf RM, Adams KF (2002). Outcomes of a Prospective Trial of Intravenous Milrinone for Exacerbations of Chronic Heart Failure (OPTIMECHF) Investigators. Short-term intravenous milrinone for acute exacerbation of chronic heart failure: a randomized controlled trial. JAMA.

[11] Burger AJ, Horton DP, LeJemtel T (2002). Effect of nesiritide (B-type natriuretic peptide) and dobutamine on ventricular arrhythmias in the treatment of patients with acutely decompensated congestive heart failure: the PRECEDENT study. Am Heart J.

[12] Abraham WT, Adams KF, Fonarow GC (2005). In-hospital mortality in patients with acute decompensated heart failure requiring intravenous vasoactive medications: an analysis from the acute decompensated heart failure national registry (ADHERE). J Am Coll Cardiol.

[13] Ho KK, Pinsky JL, Kannel WB, Levy D (1993). The epidemiology of heart failure: the Framingham Study. J Am Coll Cardiol.

[14] Al Suwaidi J, Bener A, Hajar HA, Numan MT (2004). Does hospitalisation for congestive heart failure occur more frequently in Ramadan: a population- based study (1991–2001). Int J Cardiol.

[15] Al Suwaidi J, Asaad N, Al-Qahtani A, Al-Mulla AW, Singh R, Albinali HA (2012). Prevalence and outcome of Middle-eastern Arab and South Asian patients hospitalized with heart failure: insight from a 20-year registry in a Middle-eastern country (1991–2010). Acute Card Care.

[16] Al Suwaidi J, Al-Qahtani A, Asaad N, Al-Mulla AW, Singh R, Albinali HA (2012). Comparison of women versus men hospitalized with heart failure (from a 20-year registry in a middle-eastern country 1991–2010). Am J Cardiol.

[17] McKee PA, Castelli WP, McNamara PM (1971). The natural history of congestive heart failure: the Framingham Study. N Engl J Med.

[18] Yancy CW, Lopatin M, Stevenson LW (2006). Clinical presentation, management, and in-hospital outcomes of patients admitted with acute decompensated heart failure with preserved systolic function: a report from the Acute Decompensated Heart Failure National Registry (ADHERE) database. J Am Coll Cardiol.

[19] Fonarow GC, for the ADHERE Scientific Advisory Committee (2003). The Acute Decompensated Heart Failure National Registry (ADHERE): opportunities to improve care of patients hospitalized with acute decompensated heart failure. Rev Cardiovasc Med.

[20] Shah MR, Hasselblad V, Stinnett SS (2001). Hemodynamic profiles of advanced heart failure: Association with clinical characteristics and long-term outcomes. J Card Fail.

[21] Stevenson LW, Massie BM, Francis GS (1998). Optimizing therapy for complex or refractory heart failure: A management algorithm. Am Heart J.

[22] Forman DE, Butler J, Wang Y (2004). Incidence, predictors at admission, and impact of worsening renal function among patients hospitalized with heart failure. J Am Coll Cardiol.

[23] Stevenson LW, Nohria A, Mielniczuk L (2005). Torrent or torment from the tubules? Challenge of the cardiorenal connections. J Am Coll Cardiol.

[24] Cleland JGF, Swedberg K, Follath F (2003). The EuroHeart Failure survey programme—A survey on the quality of care among patients with heart failure in Europe: Part 1: Patient characteristics and diagnosis. Eur Heart J.

[25] Goldberg LI (1974). Dopamine: clinical uses of an endogenous catecholamine. N Engl J Med.

[26] Leier CV (1996). Positive inotropic therapy: an update and new agents. Curr Probl Cardiol.

[27] Felker GM, Benza RL, Chandler AB (2003). Heart failure etiology and response to milrinone in decompensated heart failure: results from the OPTIME-CHF study. J Am Coll Cardiol.

[28] Young JB (1996). Evolv ing concepts in the treatment of heart failure: should new inotropic agents carry promise or paranoia?. Pharmacotherapy.

[29] Sasayama S (1992). What do the newer inotropic drugs have to offer?. Cardiovasc Drugs Ther Feb.

[30] Packer M, Carver JR, Rodeheffer RJ (1991). Effect of Oral Milrinone on mortality in severe chronic heart failure. N Engl J Med.

[31] Packer M, Leir CV (1987). Survival in congestive heart failure during treatment with drugs with positive inotropic actions. Circulation.

[32] Elis A, Bental T, Kimchi O (1998). Intermittent dobutamine treatment in patients with chronic refractory congestive heart failure: a randomized, double-blind, placebo-controlled study. Clin Pharmacol Ther.

[33] Oliva F, Latini R, Politi A (1999). Intermittent 6-month low-dose dobutamine infusion in severe heart failure: DICE multicenter trial. Am Heart J.

[34] O’Connor CM, Gattis WA, Uretsky BF (1999). Continuous intravenous dobutamine is associated with an increased risk of death in patients with advanced heart failure: insights from the Flolan International Randomized Survival Trial (FIRST). Am Heart J.

[35] Coletta AP, Cleland JG, Freemantle N (2004). Clinical trials update from the European Society of Cardiology Heart Failure meeting: SHAPE, BRING-UP 2 VAS, COLA II, FOSIDIAL, BETACAR, CASINO and meta-analysis of cardiac resynchronisation therapy. Eur J Heart Fail.

[36] Cleland JG, Ghosh J, Freemantle N (2004). Clinical trials update and cumulative meta-analyses from the American College of Cardiology: WATCH, SCD HeFT, DINAMIT, CASINO, INSPIRE, STRATUS-US, RIO-Lipids and cardiac resynchronisation therapy in heart failure. Eur J Heart Fail.

[37] Thackray S, Easthaugh J, Freemantle N (2002). The effectiveness and relative effectiveness of intravenous inotropic drugs acting through the adrenergic pathway in patients with heart failure-a meta-regression analysis. Eur J Heart Fail.

[38] Silver MA, Horton DP, Ghali JK (2002). Effect of nesiritide versus dobutamine on short-term outcomes in the treatment of patients with acutely decompensated heart failure. J Am Coll Cardiol.

[39] Pflugfelder PW, O’Neill BJ, Ogilvie RI (1991). A Canadian multicentre study of a 48 h infusion of milrinone in patients with severe heart failure. Can J Cardiol.

[40] Reassessing treatment of acute heart failure syndromes: the ADHERE Registry Mihai Gheorghiade1* and Gerasimos Filippatos2 European Heart Journal Supplements (2005) 7 (Supplement B), B13–B19. doi:10.1093/eurheartj/sui008

[41] Johnson D, Jin Y, Quan H, Cujec B (2003). Beta-blockers and angiotensinconverting enzyme inhibitors/receptor blockers prescriptions after hospital discharge for heart failure are associated with decreased mortality in Alberta, Canada. J Am Coll Cardiol.

